# Supplementary figures and images for: Development of leptospiral virulence-modifying protein detection assay: implications for pathogenesis and diagnostic test development
Source: Microbiol Spectr. 2025 Sep 29;13(11):e00018-25. doi: 10.1128/spectrum.00018-25 (PMC12584635; doi:10.1128/spectrum.00018-25)

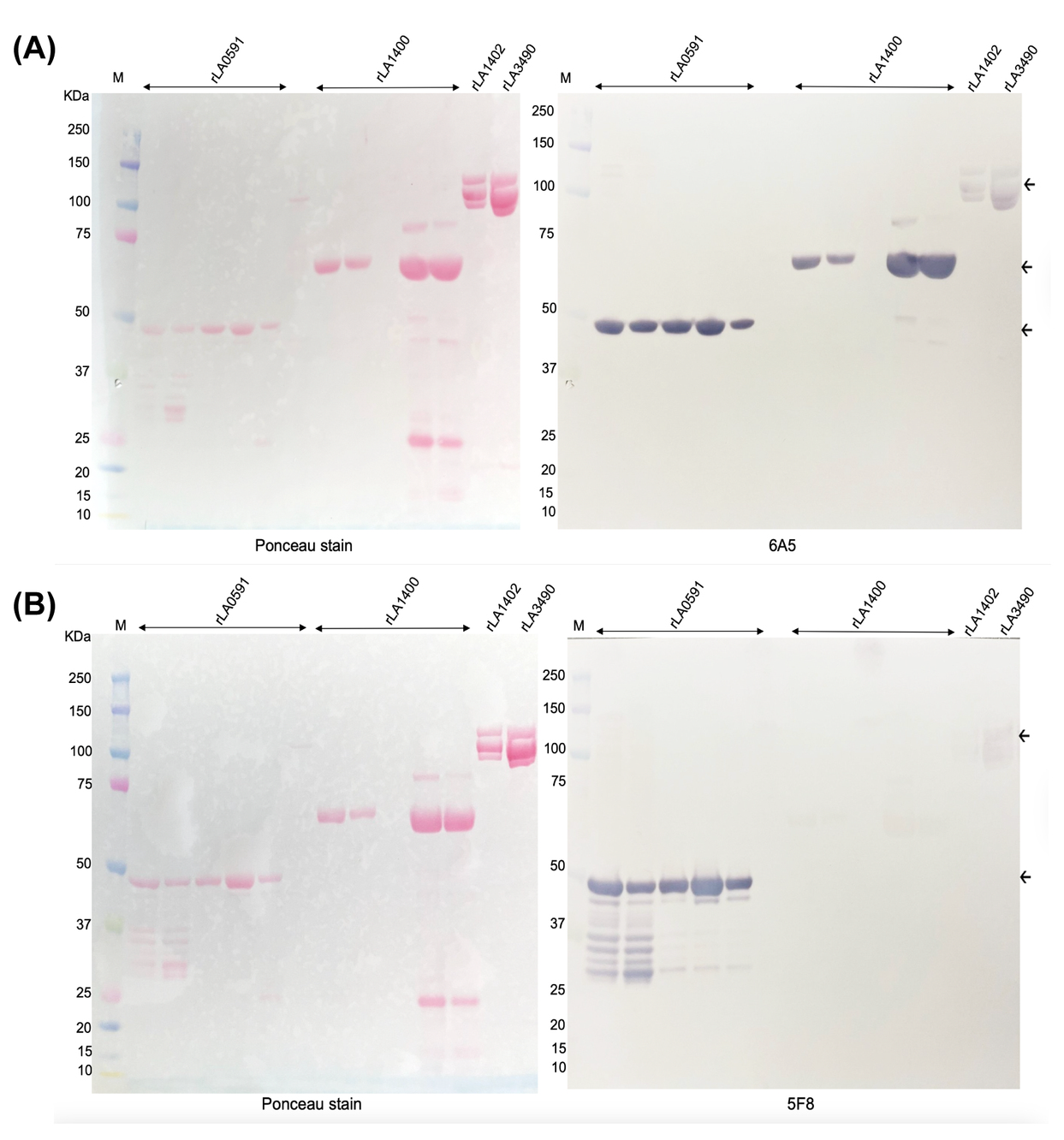

Supplement: Fig. S1 — Purity and immunoreactivity of rVM proteins. [file spectrum.00018-25-s0001.tiff]

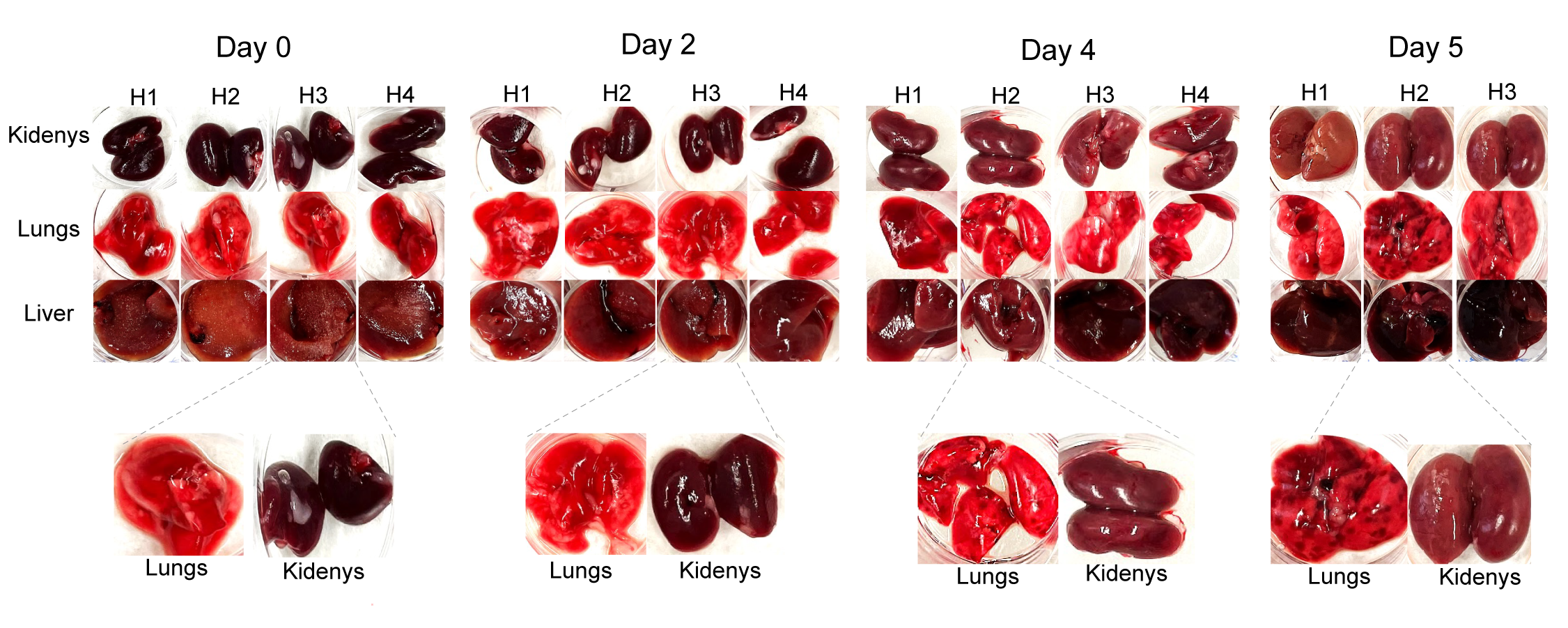

Supplement: Fig. S2 — Clinical progression in hamsters following L. interrogans serovar Copenhageni strain Fiocruz L1-130 infection at the time points. [file spectrum.00018-25-s0002.tif]
