# Supplementary material for: Development of leptospiral virulence-modifying protein detection assay: implications for pathogenesis and diagnostic test development
Source: Microbiol Spectr. 2025 Sep 29;13(11):e00018-25. doi: 10.1128/spectrum.00018-25 (PMC12584635; doi:10.1128/spectrum.00018-25)
Supplement: Supplemental material — Supplemental figure captions. [file spectrum.00018-25-s0003.docx]

**SUPPLEMENTARY FIGURE DATA LEGEND**

**Supplementary Figure 1.** **Purity and immunoreactivity of rVM proteins.** Western blot analysis of recombinant VM proteins (rLA0591, rLA1400, rLA1402, and rLA3490) probed with monoclonal antibodies 6A5 **(A)** and 5F8 **(B)**. Recombinant VMPs were separated by SDS-PAGE, transferred to membranes, and detected using 6A5 and 5F8 antibodies to confirm specificity and reactivity. Arrows show the rVM proteins.

**Supplementary Figure 2. Clinical progression in hamsters following Leptospira interrogans serovar Copenhageni strain Fiocruz L1-130 infection at time points.** Hamsters were observed at specific intervals post-infection to evaluate the development of clinical signs linked to leptospirosis. Clinical symptoms worsened over time, and gross pathological changes became evident in major organs such as the kidneys, liver, and lungs, consistent with interstitial nephritis, hepatic inflammation, and pulmonary hemorrhage. The lower panel presents enlarged, representative images of the lungs and kidneys from a single hamster in each experimental group. Notably, hamster H4 succumbed to infection on day 5 before pathological assessment.
